# Book Notice

**Published:** 1884-06

**Authors:** 


### BOOK NOTICE.

### Notes on Dental Practice by Henry C. Quinby, Licentiate in Dental Surgery of the Royal College of Surgeons in Ireland, and Member of the Odontological Societies of New York and London--With Illustrations.

In the preface the author says:

"It has not been my purpose in this work to attempt to wade in the deep waters of physiological and pathological research; but rather to confine myself as strictly as a clear explanation of my meaning will permit to the treatment of abnormal conditions of the teeth."

The first chapter is devoted to the temporary teeth, and gives many valuable suggestions and directions as to their care and management.

The second chapter to the fifth inclusive discusses the care and management of the permanent teeth. In the second chapter the importance of early and frequent attention to them is enforced; the principles of filling are considered. The detail of the explanation pertains more, however, to peculiar and exceptional cases than to those of common and every day occurrence.

Peculiar and anomolous affections of the teeth are also considered.

Chapter third; extraction as a means of preventing decay. In this chapter the author advocates the early removal of the first permanent molars for the correction of many irregularities. Indeed, he seems to recommend this practice in the large majority of cases. This is a subject upon which there is considerable diversity of opinion and practice everywhere.

Chapter fourth--Irregularities. The proper treatment of irregularities after extraction is well considered in this chapter. Quite as much is condensed in this chapter as will anywhere else be found in the same space.

The appliances presented all seem to be well adapted to the purpose for which they are designed. The omission of any reference to the "Coffin plate" seems singular, especially to those who have so successfully used it.

Chapter five—"Treatment of Adult Teeth." In this are given good practical suggestions on filling teeth. The criticism made upon the time and care devoted to large and difficult operations are hardly in accord with the views of many of our best operators, on this side of the water, at least. There are some, however, who are in full sympathy with the views of Dr. Quinby.

The case given on page 133 was an extreme one, and this mode of procedure in its treatment would be condemned by every well-informed, experienced, and discreet operator.

Chapter six--Amalgam. In this are printed the views of those who advocate the general use of amalgam as a material for filling teeth.

Chapter seven—Describe some practicable methods of inserting crowns on natural roots.

Chapter eighth--Gutta Percha for Impressions.

The work as a whole is a good one, and its examination will be profitable to any practitioner. It should be in the hands of every one. It is published by the S. S. White Dental Manufacturing Co. It is in form and make-up, characteristic of everything issuing from this house—about perfect.

